# Supplementary material for: Minimal metabolic pathway structure is consistent with associated biomolecular interactions
Source: Mol Syst Biol. 2014 Jul 1;10(7):737. doi: 10.15252/msb.20145243 (PMC4299494; doi:10.15252/msb.20145243)
Supplement: Supplementary file 6 — Supplementary Information [file msb0010-0737-sd6.pdf]

Bordbar et al. Minimal metabolic pathway structure is consistent with associated macromolecular interactions

Supplementary Material Contents

Supplementary Material 3

1. Comparison of MinSpan and Convex Optimization Methods on a Simplified Model and the *E. coli* core Model 3
2. Protein-Protein Interactions Are Conserved in Metabolic Pathways 4
3. Correlation analysis results 4
4. Criteria and full results for predicting transcriptional regulation under 51 environmental shifts ... 5
5. 28 transcription factors are associated with the anaerobic shift 6
6. In depth analysis of predicted transcriptional regulatory changes of well-studied nutrient shifts . 6
7. Analysis on the conservation of metabolic pathway structure across *E. coli* and *S. cerevisiae* 7
8. Method for calculating alternate optima MinSpan and assessment of differences 8

Supplementary Tables 11

Table S1: *E. coli* growth conditions 11

Table S2: Coverage and Area Under Curve of ROC Curve Values for Correlation Analysis........ 12

Table S3: Statistical significance of differences in AUC of ROC curves 13

Table S4: Coverage and Area Under Curve of ROC Curve Values for Correlation Analysis (KEGG subset) 14

Table S5: Statistical significance of differences in AUC of ROC curves (KEGG subset) 15

Table S6: Coverage and Area Under Curve of ROC Curve Values for Correlation Analysis (BioCyc subset) 16

Table S7: Statistical significance of differences in AUC of ROC curves (BioCyc subset)........ 17

Table S8: Coverage and Area Under Curve of ROC Curve Values for Correlation Analysis (GO subset) 18

Table S9: Statistical significance of differences in AUC of ROC curves (GO subset) 19

Table S10: Coverage and Area Under Curve of ROC Curve Values for Correlation Analysis (MinSpan subset) 20

Table S11: Statistical significance of differences in AUC of ROC curves (MinSpan subset) 21

Table S12: Coverage and Area Under Curve of ROC Curve Values for Correlation Analysis (Union)..... 22

Table S13: Statistical significance of differences in AUC of ROC curves (Union) 23

Table S14: Primary Literature Sources for verified transcription factor activities........ 24

Supplementary Figures 25

Figure S1: 25

Figure S2: 26

Figure S3: 27

Figure S4: 28

Figure S5: 29
Figure S6: 30
Figure S7: 31
Figure S8: 32
Figure S9: 33
Figure S10: 34
Figure S11: 35
Figure S12: 36
Figure S13: 37
References 38

Supplementary Material

1. Comparison of MinSpan and Convex Optimization Methods on a Simplified Model and the *E. coli* core Model

A toy model is presented in the main text Figure 1 and Figure S1A. The model consists of fourteen metabolites (m = 14) and 18 biochemical reactions (n = 18). This results in a stoichiometric matrix that is (14 x 18) in size and has a null space with dimension of 4. Thus, there are four MinSpan pathway vectors that span the space (Figure S1B). On the other hand, utilizing convex optimization methods, (Extreme Pathways (ExPas) (X3 software) and Elementary Flux Modes (EFMs) (Metatool 5.1)) we find 8 total pathways for each method though not all 8 pathways are the same (Figure S1C and S1D).

Next, we highlight the MinSpan pathways and ExPas of the metabolic model for *E. coli* core metabolism (Orth et al., 2009) in Figure S2 to further aid biological conceptualization of the MinSpan pathways, as well as contrast them with previous convex optimization methods. The *E. coli* core model is comprised of: Glycolysis, Pentose Phosphate Pathway, Citric Acid Cycle (TCA), Glyoxylate Cycle, Anapleurotic Reactions, Fermentation reactions, and a simplified Nitrogen Metabolism for Glutamine/Glutamate. The network is comprised of 72 metabolites and 95 metabolic reactions. Under minimal glucose aerobic conditions, the null space of the stoichiometric matrix has 23 dimensions, resulting in 23 MinSpan pathways (Figure S2A). The MinSpan pathways are hierarchically clustered by reaction usage and fall into broad categories including: glycolysis, anaplerotic pathways, fermentation, different uses of TCA, and pentose phosphate pathway. There are multiple glycolysis pathways representing multiple ways to mass-balance co-factor usage. In addition, one of the dimensions is a Type III loop which is a thermodynamically infeasible steady-state pathway. More information about Type III loops can be found here (Palsson, 2006). The MinSpan pathways utilize an average of 20.8 reactions per pathway.

Next we calculated the ExPas for this core model as the network is still small enough for convex analysis. The ExPas are also hierarchically clustered, but into 50 groups as there are 16690 pathways (Figure S2B). The group size and average reaction usages are also shown. The colors highlighting MinSpan pathways in Figure S2A are used in Figure S2B to show where the MinSpan pathways fall into the ExPa clustering groups. The entire bottom half of the ExPa clusters utilize the Pentose Phosphate Pathway thus only one MinSpan pathway (highlighted in yellow) falls into that broad category. All ExPa's can be reproduced by combinations of MinSpan pathways as the MinSpan is a linear basis for the null space.

In these two small examples, we see that there are many more ExPas/EFMs as MinSpan pathways. As model size increases, the number of calculated MinSpan pathways is always the dimension of the null space which scales linearly with the size of the stoichiometric matrix. Extreme pathways and elementary flux modes enumerate all pathways which scale exponentially with model size. It has been estimated that an older *E. coli* model (iJR904) has 10^{18} extreme pathways and the human network (Recon 1) has 10^{29} extreme pathways (Yeung, 2007). Thus, it is computationally intractable to calculate ExPas and EFMs for any genome-scale network and convex optimization is only applicable to small networks, such as the *E. coli* core metabolic

network. However, the MinSpan approach can be completed for larger models as demonstrated in the main text for the current *S. cerevisiae* and *E. coli* models.

2. Protein-Protein Interactions Are Conserved in Metabolic Pathways

One of the metrics utilized to determine biological relevance of MinSpan pathways is the conservation of yeast two-hybrid (Y2H) protein interactions. There are very few Y2H protein interactions in yeast metabolism and to our knowledge there has not been a comprehensive assessment of whether or not Y2H interactions are conserved within metabolic pathways.

There are 48 known pairs of proteins that have Y2H protein interactions in yeast metabolism, as defined by the scope of iMM904. We first determined how many of the protein interaction pairs were in metabolic pathways as defined by the four pathway types discussed in the main text: KEGG Modules (4 pairs), YeastCyc (25 pairs), Gene Ontology (47 pairs), and MinSpan (37 pairs) (Figure S3, red lines). 22 Y2H protein interaction pairs were consistently present in YeastCyc, Gene Ontology, and MinSpan pathways. Examples of protein interaction pairs that appeared in YeastCyc, Gene Ontology, and MinSpan include: 1) YPR069C and YLR146C which catalyze the adjacent metabolic reactions spermidine and spermine synthase in polyamine biosynthesis (http://pathway.yeastgenome.org/YEAST/NEW-IMAGE?type=PATHWAY&object=POLYAMSYN-YEAST-PWY), 2) YNR012W and YHR128W which catalyze the adjacent metabolic reactions uracil phosphoribosyltransferase and uridine kinase in pyrimidine ribonucleotide salvage pathways (http://pathway.yeastgenome.org/YEAST/NEW-IMAGE?type=PATHWAY&object=YEAST-RNT-SALV), and 3) YER090W and YKL211C which catalyze anthranilate synthase and indole-3-glycerol phosphate synthase in tryptophan biosynthesis (http://pathway.yeastgenome.org/YEAST/NEW-IMAGE?type=PATHWAY&object=TRPSYN-PWY).

To determine whether protein interactions pairs are enriched, we generated 10,000 lists of 48 random protein pairs from the metabolic proteins in iMM904. For each random list, we determined the number of random protein interaction pairs that were in metabolic pathways (Figure S3, blue histograms). We found that the real Y2H pairs were highly enriched in all metabolic pathways (for all four pathway types, $p < 1e-4$, empirical test) lending support to the notion that Y2H protein interactions are in fact conserved in metabolic pathways. The factor of enrichment, as compared to the median of the 10,000 random lists, was: 3.36x for MinSpan, N/A for KEGG (median of random lists = 0), 25x for YeastCyc, and 1.68x for Gene Ontology.

3. Correlation analysis results

The values for coverage (number of interactions, x-axis) and accuracy (y-axis) of Figure 2 of the main text are shown in Table S2. The p-values for the differences in ROC curves is presented in Table S3. The receiver operating characteristic (ROC) and precision-recall (PR) curves are shown in Figure S4. It is important to note that PR curves are primarily used for comparing information retrieval algorithms, which ignore true negative results. In this study, we are looking for how representative a metabolic pathway is to its underlying biomolecular interactions, not at how well interactions can be retrieved. Thus, true negative results are of equal importance as true

positives. As such, the ROC curve is a better measure than the PR curve. We have nonetheless included the PR curves to illustrate that the best ROC curve is often the best PR curve as well.

Calculation of these values is described in the Materials and Methods. For all three biomolecular interaction types, MinSpan is the most representative by varying degrees. MinSpan has more coverage than traditional pathway databases (KEGG, YeastCyc, and EcoCyc). Gene Ontology has more coverage than MinSpan due to the Biological Processes ontology of Gene Ontology having a larger scope than just metabolic pathways. Still, MinSpan is more representative. By definition, RandSpan and MaxSpan are less sparse linear bases for the null space of the two metabolic models than MinSpan. With a less sparse matrix, the chance of a significant pairwise correlation between two genes or two proteins is expected to increase. This should lead to a more total coverage but a lower accuracy, which is seen in the results.

As the different pathway databases and MinSpan cover different portions of metabolism, the total number of possible pair-wise interactions to be calculated differs. This means that a direct comparison cannot be made. Further, the intersection of all pathway databases is so few interactions, that a comparison for that region is not characteristic of all of metabolism. Instead, we tested each pathway database for the interactions in a particular database, as well as the union of all interactions. When there was a missing interaction, a correlation of 0 was used. Albeit an artificial introduction which skews results, this is the only way to do comparisons across the same subset. The artificial introduction typically decreases the ROC AUC, but actually increases the ROC AUC for positive genetic interactions as there are very few actual positive genetic interactions between metabolic genes.

In general, MinSpan was more representative of the biomolecular interactions. Across the 30 tested subsets of differing number of interactions, MinSpan was statistically more representative in 15 cases and was statistically less representative in only one case (intermediate transcriptional regulation for the EcoCyc subset). The ROC and PR curves for all cases and the ROC AUC values with p-values is presented in Tables S4-S13 and Figures S6-9.

4. Criteria and full results for predicting transcriptional regulation under 51 environmental shifts

To determine the accuracy of transcriptional regulation predictions by constraint-based models and MinSpan, we collected the known regulatory interactions from EcoCyc and literature (see Table S14). As described in the Material and Methods, prediction of a transcription factor's involvement in a nutrient shift was determined by hypergeometric enrichment ($p < 0.05$) of the transcription factor in the significantly changed MinSpan coefficients for that shift. For the aerobic/anaerobic shift, we completed a more extensive curation of the literature to determine the transcription factors involved in the environmental shift. The rationale and list of papers for the anaerobic shift are presented in the next section.

For a particular shift, the significant enrichments were compared to the available knowledge (e.g. EcoCyc and literature) to determine the True Positive (TP) and False Negative (FN) predictions. Specificity of the MinSpan prediction was determined by testing for enrichment based on a

binomial distribution ($p < 0.05$) of the TPs and FNs. Sensitivity was determined based on the Miss Rate (= FN/(TP+FN)). High specificity and sensitivity was deemed a correct prediction (37 total). High specificity but low sensitivity was deemed a marginal prediction (8 total). Low specificity, regardless of sensitivity was deemed a bad prediction (6 total).

A list of the MinSpan predictions, known EcoCyc and literature TF interactions, and their comparison is provided in Supplementary File (tfShiftResults.xlsx).

5. 28 transcription factors are associated with the anaerobic shift

28 transcription factors were determined to be associated with the anaerobic/aerobic shift. All known electron donors and acceptors, terminal reductases and oxidases, and respiratory dehydrogenases were identified for *E. coli*'s respiratory chain in the literature (Unden and Dunnwald, 2008). TFs that either sensed the electron donor and acceptors or were strong regulators of the reductases, oxidases, and dehydrogenases comprised 25 of the 28 TFs included in our list (ArcA (Compan and Touati, 1994), Cra (Ramseier et al., 1995), CysB (Hryniewicz and Kredich, 1994), DcuR (Salmon et al., 2003), FhlA (Schlensog and Bock, 1990), Fnr (Unden and Trageser, 1991), Fur (D'Autreaux et al., 2002), GlpR (Yang et al., 1997), GntR (Izu et al., 1997), HycA (Sauter et al., 1992), IscR (Schwartz et al., 2001), KdgR (Murray and Conway, 2005), LldR (Aguilera et al., 2008), MarA (Martin et al., 2002), ModE (McNicholas and Gunsalus, 2002), NadR (Kurnasov et al., 2002), NarL (Gunsalus et al., 1989), NarP (Gunsalus et al., 1989), NsrR (Bodenmiller and Spiro, 2006), NtrC (Sasse-Dwight and Gralla, 1988), OxyR (Zheng et al., 1998), PdhR (Quail and Guest, 1995), PhoP (Kasahara et al., 1992), SoxS (Demple, 1996), and TorR (Simon et al., 1994)). Three other TFs were also included based on EcoCyc annotation (Keseler et al., 2011) (AdiY, AppY, and HyfR).

6. In depth analysis of predicted transcriptional regulatory changes of well-studied nutrient shifts

Of the 51 shifts, five well studied shifts were examined in closer detail. These shifts include amino acid stimulation by arginine, leucine, and tryptophan (Cho et al., 2012), adenine stimulation (Cho et al., 2011), and the anaerobic/aerobic shift. Arginine stimulation resulted in four significantly changed pathways (Figure S10A). Two of the pathways are involved in biosynthesis of L-arginine from L-glutamate and ammonia. Both of these MinSpan pathways are regulated by ArgR. Two TFs (ArgR and GadW) are highly enriched in the four pathways ($p < 0.05$, hypergeometric test). Leucine stimulation resulted in two changed pathways associated with two enriched TFs: LeuO and Lrp (Figure S10B). The two pathways represent the biosynthetic pathway of L-leucine from pyruvate. Tryptophan supplementation significantly changed four pathways associated with three enriched TFs: TyrR, TrpR, and Cra (Figure S10C). The four pathways combine to form the biosynthetic pathway of L-tryptophan from erythrose-4-phosphate. TyrR regulates two of the pathways while TrpR regulates the final pathway in the biosynthetic pathway. Cra regulates two of the pathways implying a role in the L-tryptophan shift, but the transcription factor has not been associated with the nutrient shift in the literature. Adenine stimulation changed five pathways associated with the enriched TFs of PurR and Nac (Figure S10D). Nac has not been previously associated with adenine stimulation. The oxygen

shift is global and affects 70 MinSpan pathways that are associated with 54 of the 154 model related TFs. Of the 54 TFs, 12 are enriched in the 70 pathways (Figure S10E). To check the accuracy of *in silico* predictions, we used primary literature and gene ontology to determine 28 TFs that are well associated with the shift between aerobic and anaerobic growth referred to here as the oxygen shift (Supplementary Section 5). Twenty of the 28 TFs in the oxygen shift are among those associated with the significantly changed pathways (p = 3.5e-6, hypergeometric test) and 9 of the 12 were in the enriched group (p = 3.6e-6, hypergeometric test). MntR, AtoC, and SgrR are not associated with the shift, though MntR's regulation of mntH is co-regulated by Fur (Ikeda et al., 2005).

7. Analysis on the conservation of metabolic pathway structure across *E. coli* and *S. cerevisiae*

A characteristic of human-defined pathways is that they are often universal and conserved across multiple organisms, providing a common "language" for studying biochemistry. Though a common "language" can be advantageous, it can also be a weakness as distant organisms can be quite different, and the operation of metabolic pathways might be incorrectly represented.

As a preliminary analysis, we assessed the conservation of MinSpan pathways across the *S. cerevisiae* (Mo et al., 2009) and *E. coli* (Orth et al., 2011) models. The biochemistry of *E. coli* is better defined in literature and is recapitulated in the metabolic models. Thus, the *E. coli* model is much more complete than the *S. cerevisiae* model.

The *S. cerevisiae* null space is 332 dimensional, resulting in 332 pathways (Figure S11A). The *E. coli* metabolic model is more complete with a larger stoichiometric matrix and thus a higher dimensional null space with 750 MinSpan pathways (Figure S11B). The *S. cerevisiae* MinSpan matrix has 7455 non-zero entries in the matrix resulting in 2.26% of all entries in the matrix being non-zero. The *E. coli* MinSpan matrix is sparser with only 0.97% of entries being non-zero (15794 entries).

Even though the sizes of the matrices are quite different in both number of pathways and reactions, the global characteristics of the pathways are very similar. The majority of the MinSpan pathways contain around 20 reactions. The reaction usage, or the number of times that a reaction shows up across the pathways, also has a very similar distribution. Most reactions are typically used only once, with only a few reactions being used in a large number of MinSpan pathways.

Outside of the mathematical structure, we also assessed how similar the pathways were across the two organisms based on conservation of gene products. Though *E. coli* and *S. cerevisiae* have quite different metabolisms, they maintain a similar genetic core. An earlier assessment of the similarity in metabolic enzymes revealed that there are 271 common enzymes involving roughly 400 gene products (Jardine et al., 2002), representing less than half of the number of gene products contained each metabolic model. On the same order of similar enzymes, we found 272 gene-associated metabolic reactions that were conserved in *E. coli* and *S. cerevisiae* by matching the substrate to product conversions in the metabolic model.

We filtered the MinSpan pathways to only the 272 matching reactions and determined the similarity between the pathways for the two organisms based on a K-nearest neighbor search using the Pearson correlation as the distance metric. We found that MinSpan pathways were well conserved across *E. coli* and *S. cerevisiae* as compared to the RandSpan pathways (345% difference in correlation medians, p = 3.91e-220, Kolmogorov-Smirnov test) and random matrices with similar distributions of non-zero entries (83% difference in correlation medians, p = 2.31e-101, Kolmogorov-Smirnov test) (Figure S11C). The median Pearson correlation for the MinSpan pathways was 0.636.

Next, we compared the MinSpan conservation to human-defined pathways (Figure S11C). KEGG was excluded from this analysis as KEGG is pathways are universal and are not organism-specific. BioCyc (EcoCyc vs YeastCyc) and Gene Ontology were marginally, but significantly better conserved than MinSpan pathways (28% and 20% difference in correlation medians, p = 1.07e-7 and p = 3.74e-6 respectively, Kolmogorov-Smirnov test) but there was not a statistical difference between BioCyc and Gene Ontology conservation of metabolic pathways (7% difference in correlation medians, p = 0.213, Kolmogorov-Smirnov test). The median Pearson correlations for BioCyc and Gene Ontology were 0.815 and 0.761, respectively. Thus, MinSpan pathways are conserved compared to random matrices and pathways, but not to the degree of human-defined pathways.

The difference in conservation can be attributed to how the pathway types are built. Human-defined pathways are based on the topology of gene products in reaction networks. MinSpan pathways are constructed based on both the topology of the network and the function of metabolite flow. The presence or absence of gene products across multiple organisms does not necessitate that they operate together in a similar fashion. For example, Amador-Noquez et al. show that complementing genome annotation with isotope tracer studies is critical for determining how the TCA cycle operates differently in *Clostridium acetobutylicum* (Amador-Noquez et al., 2011), in comparison to the canonical use of the TCA cycle.

As noted in the main text, these results are preliminary as the comparison is of only two organisms that have quite differing metabolisms (Jardine et al., 2002). Further research is needed with dozens of metabolic reconstruction, both close and distant in the phylogenetic tree, to fully assess the conservation of MinSpan pathways.

8. Method for calculating alternate optima MinSpan and assessment of differences

Coleman and Pothen proved that a greedy algorithm, such as the MinSpan algorithm, yields a globally optimal sparse null space. The number of non-zero entries in a MinSpan pathway matrix is optimal and unique. However, the MinSpan algorithm is a MILP problem, and like other LP and MILP problems used in constraint-based modeling (e.g. FBA), alternate optimal solutions might exist. Thus, other alternate MinSpan pathway matrices may exist that have the same number of non-zero entries but with slightly different pathways.

To determine the differences between different alternate matrices, we formulated an algorithm that takes a completed MinSpan pathway matrix as an input and enumerates alternate optimal vectors for each pathway vector. For each pathway vector, the algorithm removes the one pathway, and then exhaustively determines different pathways with the same reaction number that still span that subspace. Thus, the output of the algorithm is a list of vectors for each column of the original, calculated MinSpan pathway matrix. Random combinations of these vectors can then produce alternate optimal MinSpan pathway matrices.

For each original MinSpan vector, we enumerate new alternate MinSpan vectors. For the jth run, the algorithm looks as follows:

$$\min(\mathbf{c}\cdot\mathbf{b})$$
$$\mathbf{c}=\mathbf{b^0}$$
$$\mathbf{b}\in\{0,1\}$$
$$\sum \mathrm{b_i}=n$$
$$\mathbf{b^0}\cdot\mathbf{b}\le n$$
$$\mathbf{B_0^T}\cdot\mathbf{b}\le\begin{bmatrix} n-1\\ \vdots \\ n-1\end{bmatrix}$$
$$\mathbf{S}\cdot\mathbf{v}=0$$
$$\mathbf{lb}\le\mathbf{v}\le\mathbf{ub}$$
$$-1000b_i\le v_i\le 1000b_i$$
$$\mathbf{x^T}\cdot\mathbf{v}\ne 0 \qquad \mathrm{z}$$

where $\mathbf{b^0}$ is the binarized version of the original MinSpan pathway vector, **b** is the binarized alternate MinSpan pathway vector that is being calculated, $\mathbf{B_0}$ is a matrix containing binarized versions of the previously determined alternates, and *n* is the total number of active reactions in that MinSpan vector. The original pathway vector is a feasible solution as to allow it to be a feasible warm up point. An alternate is chosen when any feasible solution is found that is not the original MinSpan pathway vector. The algorithm is allowed to run for 5 minutes and if no alternate is found, the algorithm moves onto the next MinSpan vector. The remaining constraints are the same as the original formulation in Materials and Methods.

We calculated alternate MinSpan for the *S. cerevisiae* and *E. coli* metabolic models. Of the 332 metabolic pathways in *S. cerevisiae*, 148 do not have any alternatives and 50 have one alternative. Of the 750 metabolic pathways in *E. coli*, 328 do not have any alternates and 152 have one alternate. The number of alternates per pathway for both models is shown in Figure S12.

Using the alternate pathways, we built 1000 MinSpan pathway matrices for each of the *S. cerevisiae* and *E. coli* metabolic models. We determined the difference between the original MinSpan pathway matrix and the 1000 alternates. Using a K-nearest neighbor search, we matched the pathways from the original matrix to an alternate matrix using the Hamming distance, or the percentage of coordinates that differ. On average for *S. cerevisiae* across the

comparison of all 1000 alternate MinSpan pathway matrices to the original, a single alternate pathway differed by 0.66% reactions, meaning that for every 10 pathways in the network, there would be one pathway, with one reaction difference. In *E. coli*, each pathway differed on average by 0.26% reactions.

We then repeated the correlation analysis (Figure 2, main text) with the 1000 pathway matrices to see how much the biological relevance results changed. We found very little difference for the correlation analysis across the 1000 pathway matrices. Especially, when comparing to the other databases (Figure 2, main text), the MinSpan (and the alternates) results were quite distinct.

For PPIs, the original *S. cerevisiae* MinSpan pathway matrix had a coverage of 4984 interactions with ROC AUC of 0.953. The 1000 alternate models had similar values (coverage: 5053 – mean, 31 – standard deviation, [4953, 5143] - range; ROC AUC: 0.9623 – mean, 0.0061 – standard deviation, [0.943, 0.9684] - range). For positive genetic interactions, the original *S. cerevisiae* MinSpan pathway matrix had a coverage of 12208 interactions with ROC AUC of 0.922. The 1000 alternate models had similar results (coverage: 12350 – mean, 79.8 – standard deviation, [12110, 12609] - range; ROC AUC: 0.8964 – mean, 0.0189 – standard deviation, [0.8318, 0.929] - range).

For transcriptional regulation in *E. coli*, the results were also very similar. For local regulation, the original results had a coverage of 9691 interactions and a ROC AUC of 0.935. The 1000 alternates had similar values (coverage: 10017.7 – mean, 108 – standard deviation, [9727, 10402] – range; ROC AUC: 0.935 – mean, 0.002 – standard deviation, [0.930, 0.940] – range). For intermediate regulation, the original results had a coverage of 10590 interactions and a ROC AUC of 0.851. For the 1000 alternates, the results were similar (coverage: 10943.3 – mean, 113.6 – standard deviation, [10640, 11342] – range; ROC AUC: 0.843 – mean, 0.0101 – standard deviation, [0.810, 0.868] – range). Finally, for global regulation, the original results had a coverage of 15013 interactions with a ROC AUC of 0.556. The alternate MinSpan pathway matrices had similar results (coverage: 15487.8 – mean, 206.7 – standard deviation, [14984, 16076] – range; ROC AUC: 0.545 – mean, 0.0069 – standard deviation, [0.526, 0.562] – range).

Supplementary Tables

Table S1: *E. coli* growth conditions

Strain	Gene ID	O_2 (+/-)	Growth Condition
wt	n/a	+	M9 + 4 g/L Glc
Δcra	b0080	+	M9 + 4 g/L Glc
ΔmntR	b0817	+	M9 + 4 g/L Glc
Δnac	b1988	+	M9 + 4 g/L Glc
wt	n/a	+	M9 + 4 g/L Glc + 20 mg/L L-Tryptophan
Δcra	b0080	+	M9 + 4 g/L Glc + 20 mg/L L-Tryptophan
wt	n/a	+	M9 + 4 g/L Glc + 10 mM Adenine
Δnac	b1988	+	M9 + 4 g/L Glc + 10 mM Adenine
wt	n/a	-	M9 + 4 g/L Glc
ΔmntR	b0817	-	M9 + 4 g/L Glc

Table S2: Coverage and Area Under Curve of ROC Curve Values for Correlation Analysis

ROC AUC	MinSpan	KEGG	BioCyc	Gene Ontology	MaxSpan	RandSpan
Protein-protein	0.958	0.691	0.890	0.953	0.802	0.865 +/- 0.051
Positive genetic	0.937*	0.520	0.800	0.670	0.502	0.611 +/- 0.0953
TR - local	0.940*	0.751	0.900	0.839	0.710	0.818 +/- 0.0323
TR - intermediate	0.853*	0.776	0.778	0.737	0.576	0.600 +/- 0.0403
TR - global	0.586	0.542	0.598	0.553	0.434	0.515 +/- 0.0876
**Total # interactions**						
Protein-protein	4984	157	1229	8777	39044	1903 +/- 942
Positive genetic	12208	186	1679	18027	79896	28708 +/- 8977
TR - local	9691	198	972	13133	177491	128790 +/- 22067
TR - intermediate	10590	343	1363	13857	180427	130983 +/- 22373
TR - global	15013	541	2162	18442	227000	166850 +/- 28492

Abbreviations: TR – transcriptional regulation

Note: RandSpan values are the mean and standard deviation of 100 randomly generated linear bases of the null space

* - MinSpan is significantly more representative based on AUC of ROC than at least two other databases

† - MinSpan is significantly less representative based on AUC of ROC than at least one other database

Table S3: Statistical significance of differences in AUC of ROC curves

**Protein-protein**						
	KEGG	BioCyc	GO	MinSpan	MaxSpan	RandSpan
KEGG	-	2.01E-01	8.32E-02	7.80E-02	4.74E-01	2.81E-01
BioCyc	-	-	2.01E-01	1.68E-01	1.36E-01	7.38E-01
GO	-	-	-	9.01E-01	1.33E-03	1.75E-01
MinSpan	-	-	-	-	8.39E-04	1.52E-01
MaxSpan	-	-	-	-	-	3.84E-01
RandSpan	-	-	-	-	-	-
**Positive Genetic**						
	KEGG	BioCyc	GO	MinSpan	MaxSpan	RandSpan
KEGG	-	6.78E-02	3.14E-01	3.16E-03	9.01E-01	6.28E-01
BioCyc	-	-	2.07E-01	1.33E-01	8.62E-04	2.23E-01
GO	-	-	-	1.47E-03	4.05E-02	6.96E-01
MinSpan	-	-	-	-	9.80E-11	2.26E-02
MaxSpan	-	-	-	-	-	4.42E-01
RandSpan	-	-	-	-	-	-
**TR - local**						
	KEGG	BioCyc	GO	MinSpan	MaxSpan	RandSpan
KEGG	-	9.30E-03	1.16E-01	2.79E-04	4.48E-01	2.15E-01
BioCyc	-	-	9.45E-02	1.81E-01	2.45E-08	1.41E-02
GO	-	-	-	2.35E-04	4.69E-05	4.99E-01
MinSpan	-	-	-	-	2.50E-19	1.71E-07
MaxSpan	-	-	-	-	-	1.24E-04
RandSpan	-	-	-	-	-	-
**TR - int**						
	KEGG	BioCyc	GO	MinSpan	MaxSpan	RandSpan
KEGG	-	9.47E-01	1.44E-01	3.38E-03	1.83E-14	1.16E-11
BioCyc	-	-	1.85E-02	3.97E-06	1.08E-29	7.54E-25
GO	-	-	-	3.30E-18	1.23E-31	4.74E-25
MinSpan	-	-	-	-	2.76E-60	1.38E-54
MaxSpan	-	-	-	-	-	3.44E-03
RandSpan	-	-	-	-	-	-
**TR - global**						
	KEGG	BioCyc	GO	MinSpan	MaxSpan	RandSpan
KEGG	-	5.38E-02	6.71E-01	1.04E-01	5.32E-05	3.14E-01
BioCyc	-	-	5.51E-04	3.40E-01	4.82E-30	1.93E-11
GO	-	-	-	1.66E-06	5.34E-52	6.55E-14
MinSpan	-	-	-	-	3.92E-60	8.86E-31
MaxSpan	-	-	-	-	-	2.43E-68
RandSpan	-	-	-	-	-	-

Table S4: Coverage and Area Under Curve of ROC Curve Values for Correlation Analysis (KEGG subset)

ROC AUC	MinSpan	KEGG	BioCyc	Gene Ontology
Protein-protein	0.845	0.691	0.737	0.805
Positive genetic	0.814	0.520	0.736	0.812
TR - local	0.954*	0.751	0.723	0.879
TR - intermediate	0.792	0.776	0.717	0.824
TR - global	0.614	0.542	0.554	0.681

* - MinSpan is significantly more representative based on AUC of ROC than at least two other databases

† - MinSpan is significantly less representative based on AUC of ROC than at least one other database

Table S5: Statistical significance of differences in AUC of ROC curves (KEGG subset)

**Protein-protein**				
	KEGG	BioCyc	GO	MinSpan
KEGG	-	8.26E-01	5.72E-01	4.29E-01
BioCyc	-	-	7.31E-01	5.72E-01
GO	-	-	-	8.26E-01
MinSpan	-	-	-	-
**Positive Genetic**				
	KEGG	BioCyc	GO	MinSpan
KEGG	-	2.44E-01	9.90E-02	9.69E-02
BioCyc	-	-	6.62E-01	6.55E-01
GO	-	-	-	9.93E-01
MinSpan	-	-	-	-
**TR - local**				
	KEGG	BioCyc	GO	MinSpan
KEGG	-	6.93E-01	4.51E-02	3.00E-04
BioCyc	-	-	1.60E-02	5.64E-05
GO	-	-	-	1.00E-01
MinSpan	-	-	-	-
**TR - int**				
	KEGG	BioCyc	GO	MinSpan
KEGG	-	1.07E-01	1.54E-01	6.61E-01
BioCyc	-	-	2.38E-03	4.03E-02
GO	-	-	-	3.23E-01
MinSpan	-	-	-	-
**TR - global**				
	KEGG	BioCyc	GO	MinSpan
KEGG	-	7.41E-01	9.46E-05	5.12E-02
BioCyc	-	-	3.58E-04	1.06E-01
GO	-	-	-	5.26E-02
MinSpan	-	-	-	-

Table S6: Coverage and Area Under Curve of ROC Curve Values for Correlation Analysis (BioCyc subset)

ROC AUC	MinSpan	KEGG	BioCyc	Gene Ontology
Protein-protein	0.870	0.610	0.890	0.939
Positive genetic	0.810	0.674	0.800	0.730
TR - local	0.882	0.659	0.900	0.887
TR - intermediate	0.697†	0.625	0.778	0.773
TR - global	0.711*	0.531	0.598	0.645

* - MinSpan is significantly more representative based on AUC of ROC than at least two other databases

† - MinSpan is significantly less representative based on AUC of ROC than at least one other database

Table S7: Statistical significance of differences in AUC of ROC curves (BioCyc subset)

**Protein-protein**				
	KEGG	BioCyc	GO	MinSpan
KEGG	-	1.63E-04	1.98E-06	6.11E-04
BioCyc	-	-	3.69E-01	7.55E-01
GO	-	-	-	2.27E-01
MinSpan	-	-	-	-
**Positive Genetic**				
	KEGG	BioCyc	GO	MinSpan
KEGG	-	2.75E-01	6.37E-01	2.40E-01
BioCyc	-	-	5.40E-01	9.35E-01
GO	-	-	-	4.87E-01
MinSpan	-	-	-	-
**TR - local**				
	KEGG	BioCyc	GO	MinSpan
KEGG	-	5.77E-07	3.05E-06	6.17E-06
BioCyc	-	-	7.50E-01	6.47E-01
GO	-	-	-	8.88E-01
MinSpan	-	-	-	-
**TR - int**				
	KEGG	BioCyc	GO	MinSpan
KEGG	-	3.99E-12	2.05E-11	1.52E-03
BioCyc	-	-	8.01E-01	1.27E-04
GO	-	-	-	3.46E-04
MinSpan	-	-	-	-
**TR - global**				
	KEGG	BioCyc	GO	MinSpan
KEGG	-	1.01E-04	5.23E-11	3.50E-23
BioCyc	-	-	6.35E-03	9.23E-12
GO	-	-	-	3.06E-05
MinSpan	-	-	-	-

Table S8: Coverage and Area Under Curve of ROC Curve Values for Correlation Analysis (GO subset)

ROC AUC	MinSpan	KEGG	BioCyc	Gene Ontology
Protein-protein	0.931*	0.620	0.895	0.917
Positive genetic	0.740	0.630	0.741	0.670
TR - local	0.924*	0.651	0.747	0.840
TR - intermediate	0.787*	0.594	0.681	0.737
TR - global	0.582*	0.525	0.554	0.553

* - MinSpan is significantly more representative based on AUC of ROC than at least two other databases

† - MinSpan is significantly less representative based on AUC of ROC than at least one other database

Table S9: Statistical significance of differences in AUC of ROC curves (GO subset)

**Protein-protein**				
	KEGG	BioCyc	GO	MinSpan
KEGG	-	9.04E-05	4.44E-11	1.93E-11
BioCyc	-	-	1.64E-02	1.19E-02
GO	-	-	-	9.01E-01
MinSpan	-	-	-	-
**Positive Genetic**				
	KEGG	BioCyc	GO	MinSpan
KEGG	-	2.43E-01	6.82E-01	2.49E-01
BioCyc	-	-	4.52E-01	9.88E-01
GO	-	-	-	4.61E-01
MinSpan	-	-	-	-
**TR - local**				
	KEGG	BioCyc	GO	MinSpan
KEGG	-	1.72E-02	7.59E-07	9.41E-15
BioCyc	-	-	1.25E-02	8.53E-08
GO	-	-	-	4.61E-03
MinSpan	-	-	-	-
**TR - int**				
	KEGG	BioCyc	GO	MinSpan
KEGG	-	7.92E-09	7.21E-20	4.55E-30
BioCyc	-	-	1.13E-04	2.86E-13
GO	-	-	-	3.65E-04
MinSpan	-	-	-	-
**TR - global**				
	KEGG	BioCyc	GO	MinSpan
KEGG	-	1.05E-05	1.69E-05	1.96E-16
BioCyc	-	-	9.16E-01	3.52E-05
GO	-	-	-	2.22E-05
MinSpan	-	-	-	-

Table S10: Coverage and Area Under Curve of ROC Curve Values for Correlation Analysis (MinSpan subset)

ROC AUC	MinSpan	KEGG	BioCyc	Gene Ontology
Protein-protein	0.958	0.577	0.836	0.953
Positive genetic	0.937	0.713	0.953	0.900
TR - local	0.940*	0.602	0.644	0.742
TR - intermediate	0.853*	0.564	0.604	0.700
TR - global	0.586*	0.519	0.555	0.557

* - MinSpan is significantly more representative based on AUC of ROC than at least two other databases

† - MinSpan is significantly less representative based on AUC of ROC than at least one other database

Table S11: Statistical significance of differences in AUC of ROC curves (MinSpan subset)

**Protein-protein**				
	KEGG	BioCyc	GO	MinSpan
KEGG	-	8.77E-06	7.85E-07	1.26E-07
BioCyc	-	-	6.50E-01	4.38E-01
GO	-	-	-	7.46E-01
MinSpan	-	-	-	-
**Positive Genetic**				
	KEGG	BioCyc	GO	MinSpan
KEGG	-	1.12E-02	7.16E-02	2.17E-02
BioCyc	-	-	4.67E-01	8.05E-01
GO	-	-	-	6.28E-01
MinSpan	-	-	-	-
**TR - local**				
	KEGG	BioCyc	GO	MinSpan
KEGG	-	2.08E-01	1.59E-05	5.21E-28
BioCyc	-	-	2.44E-03	8.23E-24
GO	-	-	-	4.63E-14
MinSpan	-	-	-	-
**TR - int**				
	KEGG	BioCyc	GO	MinSpan
KEGG	-	2.93E-03	3.87E-21	3.66E-51
BioCyc	-	-	2.17E-12	2.58E-45
GO	-	-	-	1.89E-28
MinSpan	-	-	-	-
**TR - global**				
	KEGG	BioCyc	GO	MinSpan
KEGG	-	1.12E-07	4.05E-08	6.82E-20
BioCyc	-	-	8.51E-01	1.05E-05
GO	-	-	-	2.44E-05
MinSpan	-	-	-	-

Table S12: Coverage and Area Under Curve of ROC Curve Values for Correlation Analysis (Union)

ROC AUC	MinSpan	KEGG	BioCyc	Gene Ontology
Protein-protein	0.906	0.605	0.859	0.859
Positive genetic	0.721	0.595	0.787	0.696
TR - local	0.918*	0.593	0.645	0.669
TR - intermediate	0.763*	0.557	0.634	0.609
TR - global	0.584*	0.52	0.551	0.518

* - MinSpan is significantly more representative based on AUC of ROC than at least two other databases

† - MinSpan is significantly less representative based on AUC of ROC than at least one other database

Table S13: Statistical significance of differences in AUC of ROC curves (Union)

**Protein-protein**				
	KEGG	BioCyc	GO	MinSpan
KEGG	-	1.33E-05	1.39E-05	5.03E-08
BioCyc	-	-	9.92E-01	3.18E-01
GO	-	-	-	3.13E-01
MinSpan	-	-	-	-
**Positive Genetic**				
	KEGG	BioCyc	GO	MinSpan
KEGG	-	1.57E-02	2.24E-01	1.23E-01
BioCyc	-	-	2.46E-01	4.02E-01
GO	-	-	-	7.50E-01
MinSpan	-	-	-	-
**TR - local**				
	KEGG	BioCyc	GO	MinSpan
KEGG	-	7.86E-02	1.45E-02	2.52E-27
BioCyc	-	-	4.95E-01	1.59E-21
GO	-	-	-	2.77E-19
MinSpan	-	-	-	-
**TR - int**				
	KEGG	BioCyc	GO	MinSpan
KEGG	-	1.42E-11	4.69E-06	1.45E-42
BioCyc	-	-	2.41E-02	7.08E-26
GO	-	-	-	6.45E-32
MinSpan	-	-	-	-
**TR - global**				
	KEGG	BioCyc	GO	MinSpan
KEGG	-	4.50E-10	7.29E-01	5.14E-29
BioCyc	-	-	5.41E-11	2.84E-11
GO	-	-	-	6.51E-30
MinSpan	-	-	-	-

Table S14: Primary Literature Sources for verified transcription factor activities

Shift	Transcription Factor	PMID or URL
Arabinose	FhlA	http://www.sciencedirect.com/science/article/pii/S0360319912017259
Fumarate	NarLP	16199562
Fumarate	DcuR	9765574
Fumarate	IscR	16677314
Galactose	HupAB	16258062
Lactose	HupAB	16258062
Pyruvate	MntR	http://onlinelibrary.wiley.com/doi/10.1002/0470862106.ia129/full
Xylose	RpoS	19650909
Nitrate	IscR	16677314
Glutathione	Nac	15286142
Isoleucine	TdcAR	9871012
Isoleucine	MetJ	4580268

Supplementary Figures

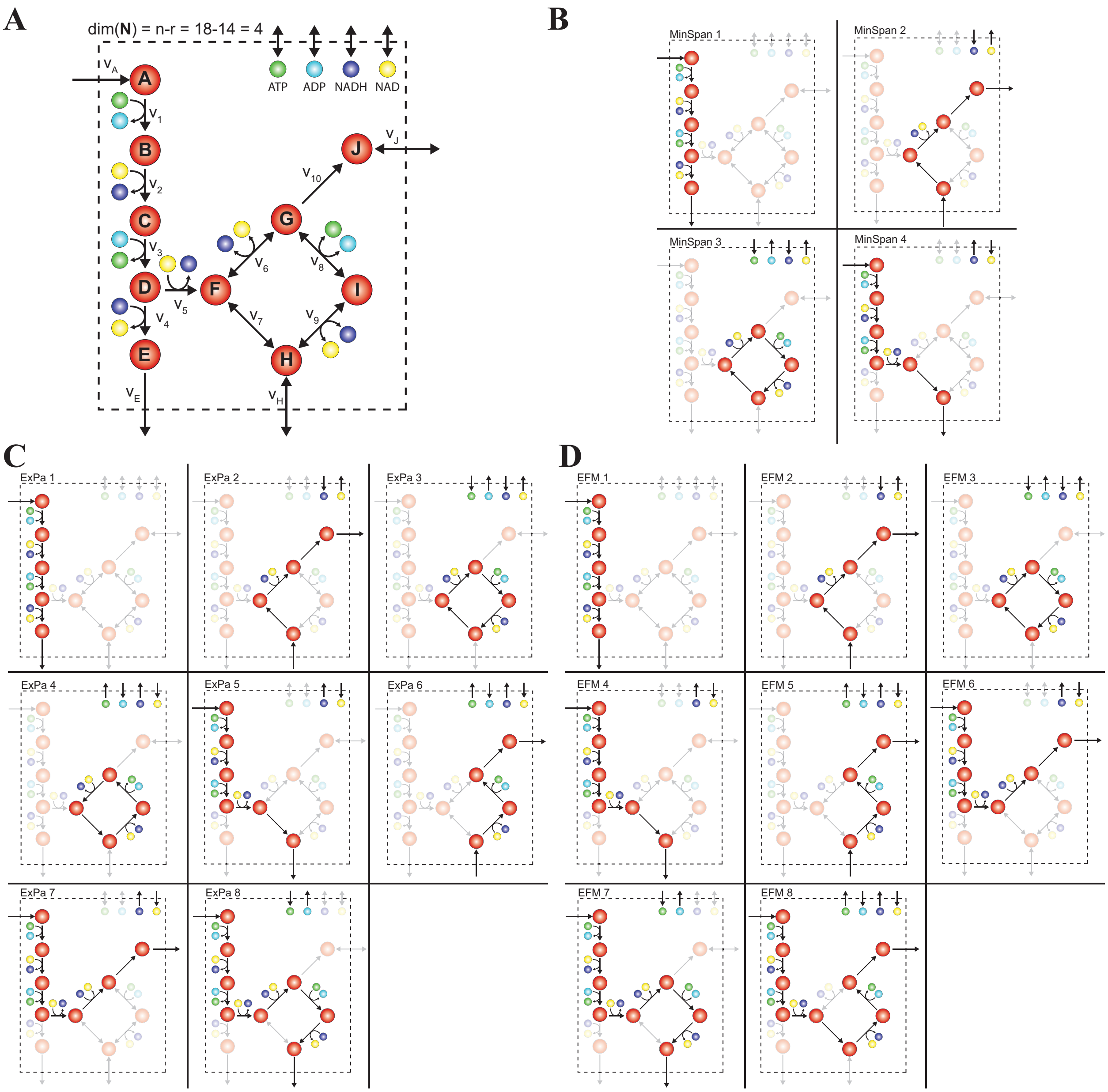

Figure S1: Pathway comparison on a toy model. (A) The toy model contains 14 moieties and 18 biochemical transformations. The stoichiometric matrix has a rank of 14, meaning that the null space is 4 dimensional. (B) The MinSpan algorithm calculates 4 pathways as the dimension of the null space is 4. They are ordered from shortest to longest pathway. (C) The toy model has 8 Extreme Pathways and (D) 8 Elementary Flux Modes.

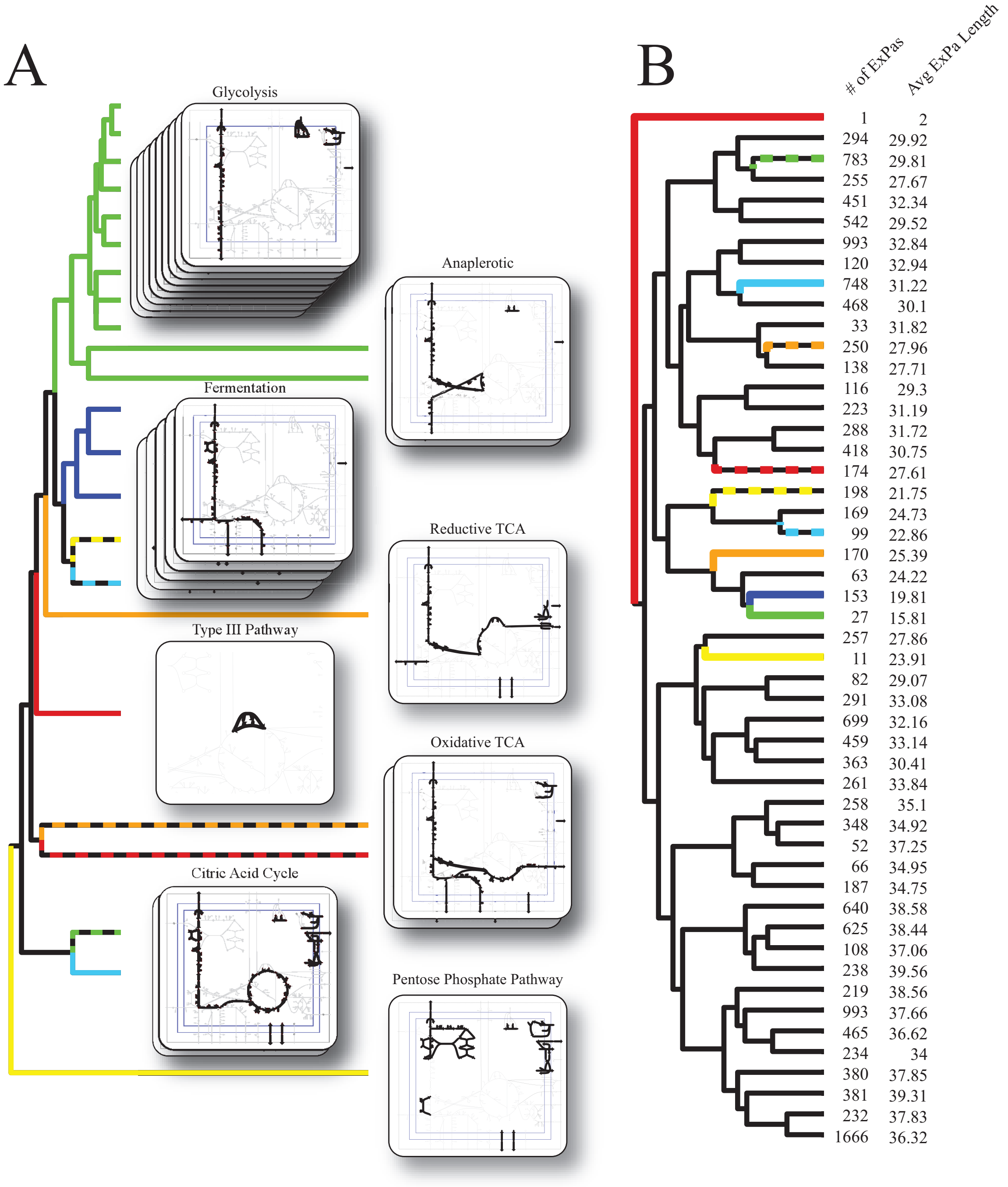

Figure S2: Comparison of MinSpan pathways and Extreme Pathways for the *E. coli* core mode are presented. (A) The 23 MinSpan pathways for this model were hierarchically clustered and placed into subsystem categories of glycolysis, anaplerotic pathways, fermentation, TCA, and pentose phosphate pathway. The MinSpan pathways are on average 20.8 reactions in length. (B) There are 16690 Extreme Pathways which were hierarchically clustered into 50 groups. The number of pathways per group and average length of pathways within that group are shown. The MinSpan pathways are a subset of the Extreme Pathways. The coloring on the dendrogram branch relates the location of the MinSpan pathways in the Extreme Pathway groups.

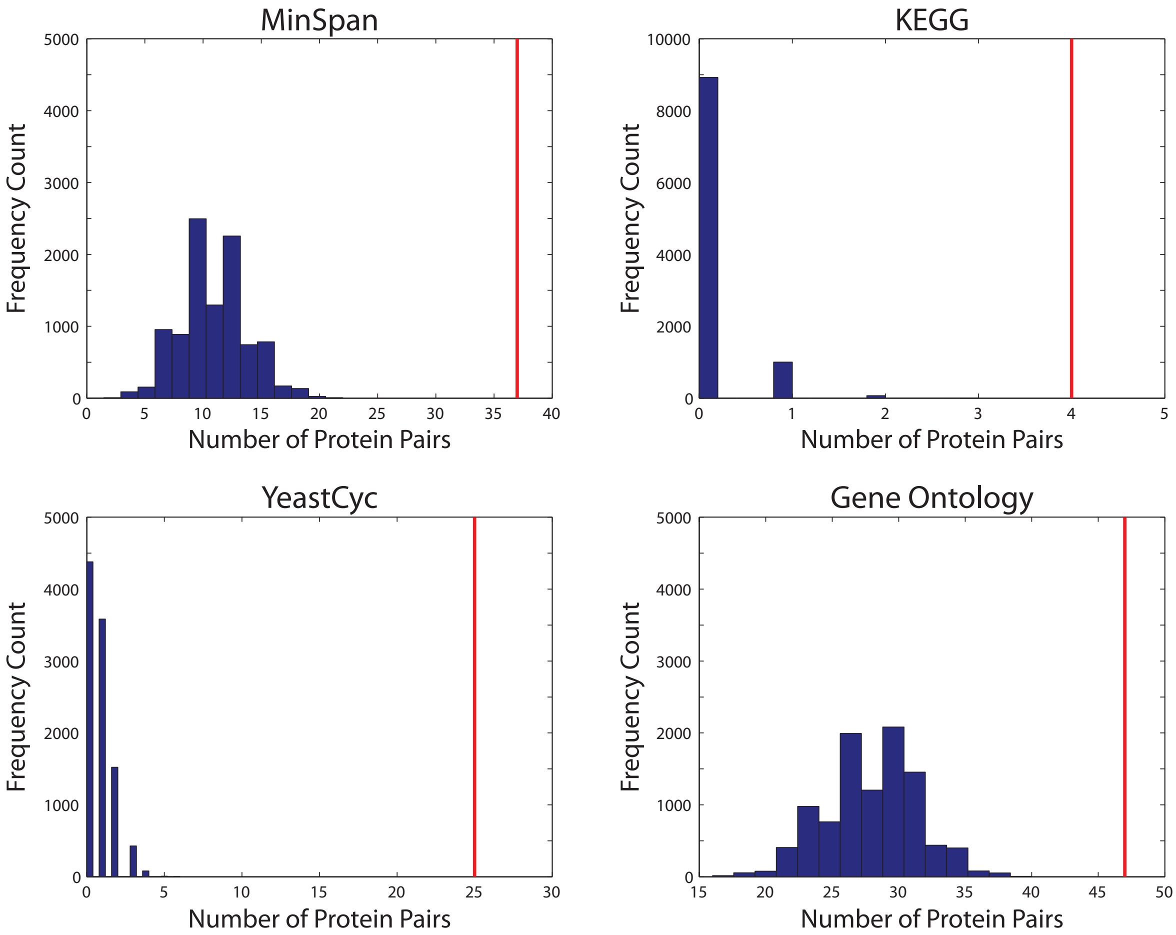

Figure S3: Yeast-2-Hybrid protein interactions are conserved in metabolic pathways. The number of Y2H interaction pairs that are in metabolic pathways is shown for each metabolic pathway type by the x-axis value of the red line. 10,000 lists of random protein pairs in yeast metabolism were generated. The number of random protein pairs that are within metabolic pathways are shown by the histogram. Enrichment of true Y2H interaction pairs in metabolic pathways is highly enriched ($p < 1e{-4}$, empirical test) for all pathway types with a high enrichment factor (3.36x for MinSpan, N/A for KEGG (median of random lists = 0), 25x for YeastCyc, and 1.68x for Gene Ontology).

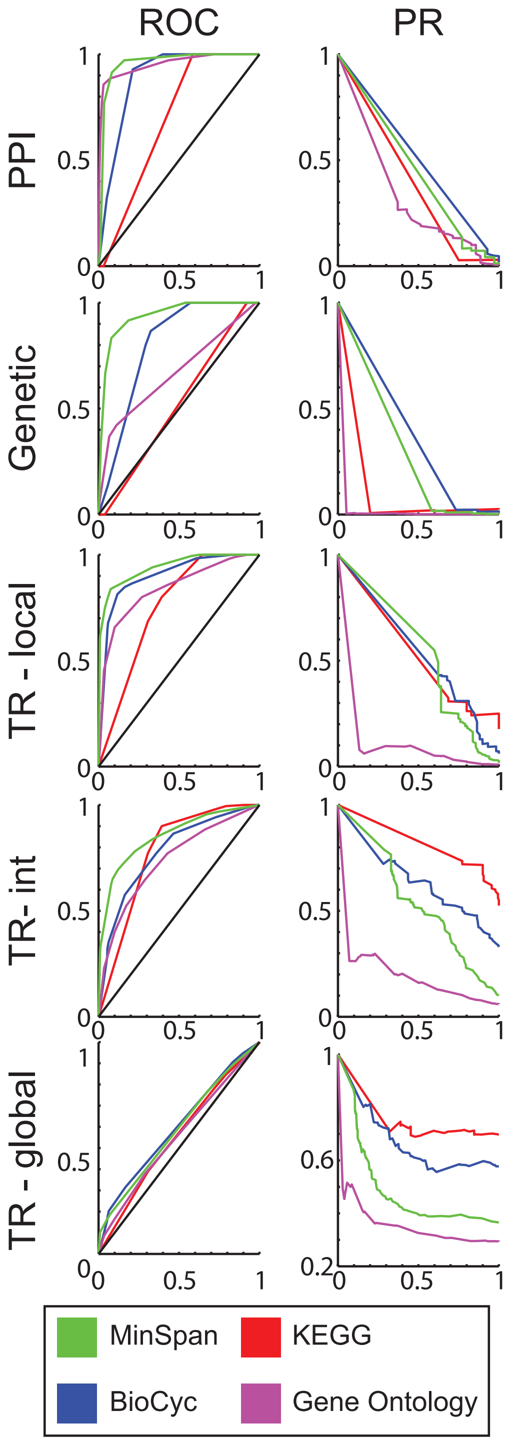

Figure S4: Receiver operating characteristic (ROC) and precision-recall (PR) curves for correlation analysis comparing metabolic pathways (MinSpan, BioCyc, and KEGG) and metabolic gene classifications (Gene Ontology) versus the known underlying biomolecular interactions (protein-protein, positive genetic, and transcriptional regulation). These curves are for the original results presented in the main text.

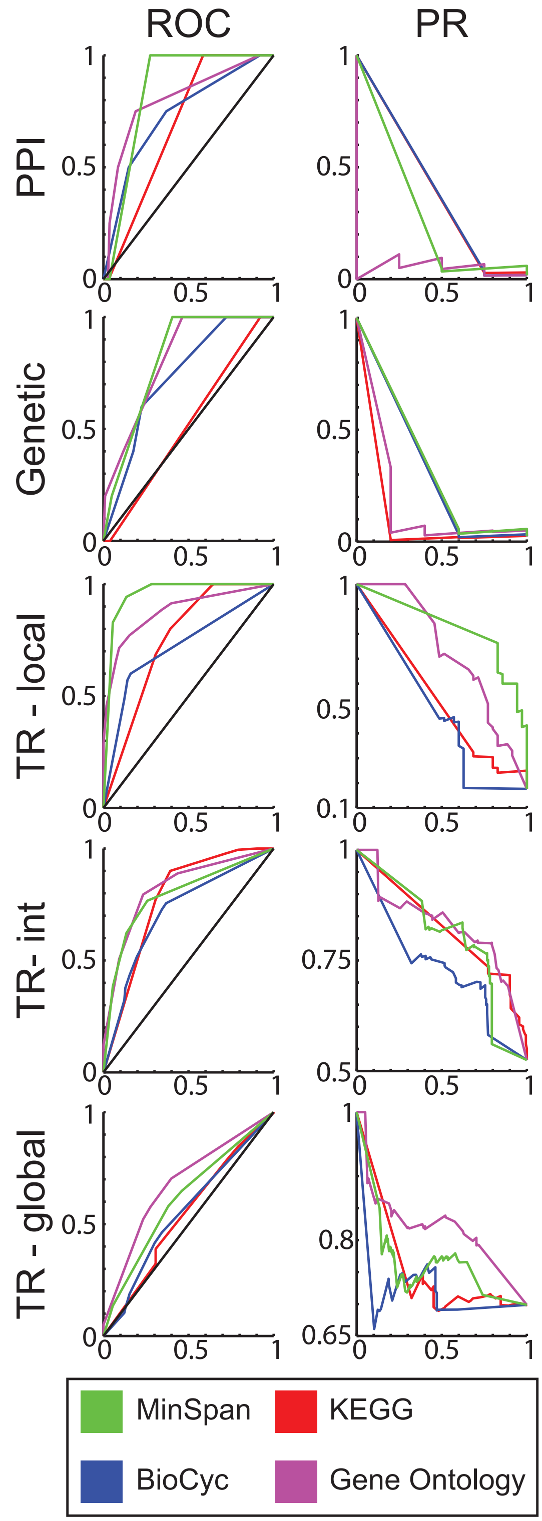

Figure S5: Receiver operating characteristic (ROC) and precision-recall (PR) curves for correlation analysis comparing metabolic pathways (MinSpan, BioCyc, and KEGG) and metabolic gene classifications (Gene Ontology) versus the known underlying biomolecular interactions (protein-protein, positive genetic, and transcriptional regulation). These curves are for the subset of interactions covered by KEGG.

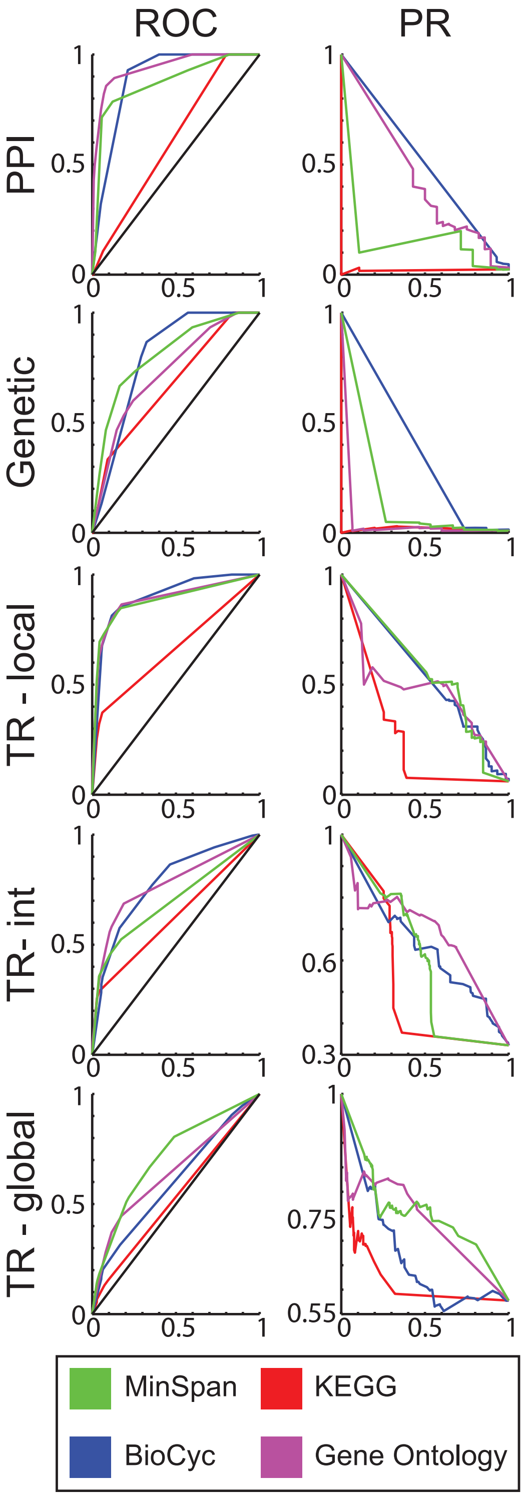

Figure S6: Receiver operating characteristic (ROC) and precision-recall (PR) curves for correlation analysis comparing metabolic pathways (MinSpan, BioCyc, and KEGG) and metabolic gene classifications (Gene Ontology) versus the known underlying biomolecular interactions (protein-protein, positive genetic, and transcriptional regulation). These curves are for the subset of interactions covered by BioCyc.

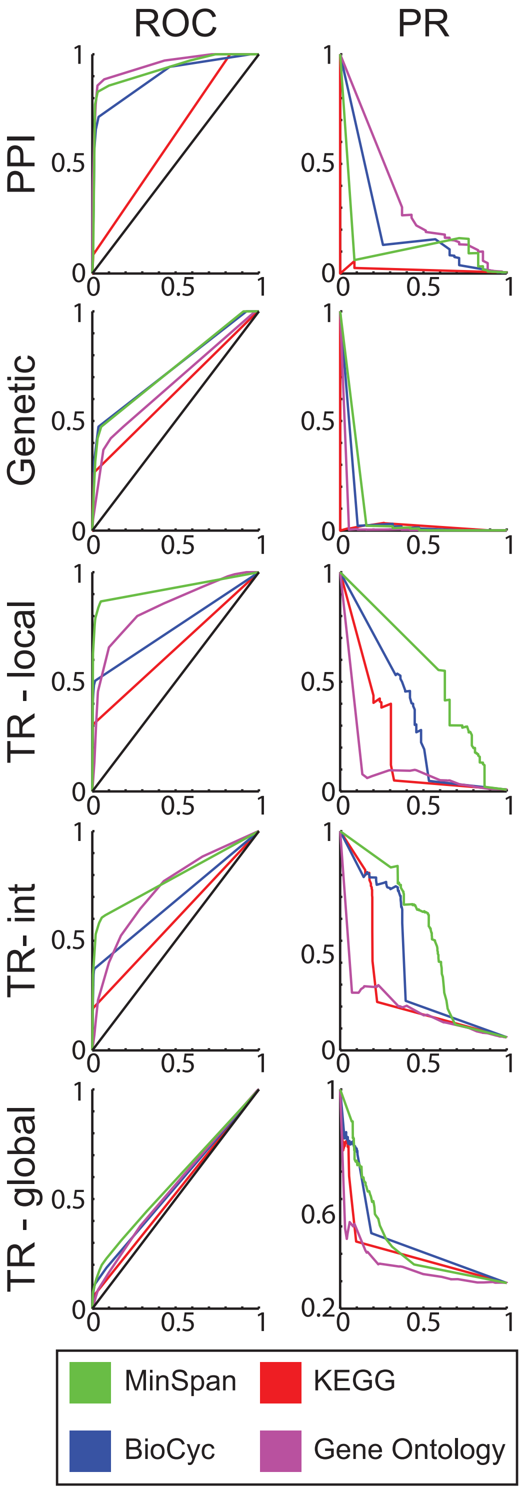

Figure S7: Receiver operating characteristic (ROC) and precision-recall (PR) curves for correlation analysis comparing metabolic pathways (MinSpan, BioCyc, and KEGG) and metabolic gene classifications (Gene Ontology) versus the known underlying biomolecular interactions (protein-protein, positive genetic, and transcriptional regulation). These curves are for the subset of interactions covered by Gene Ontology.

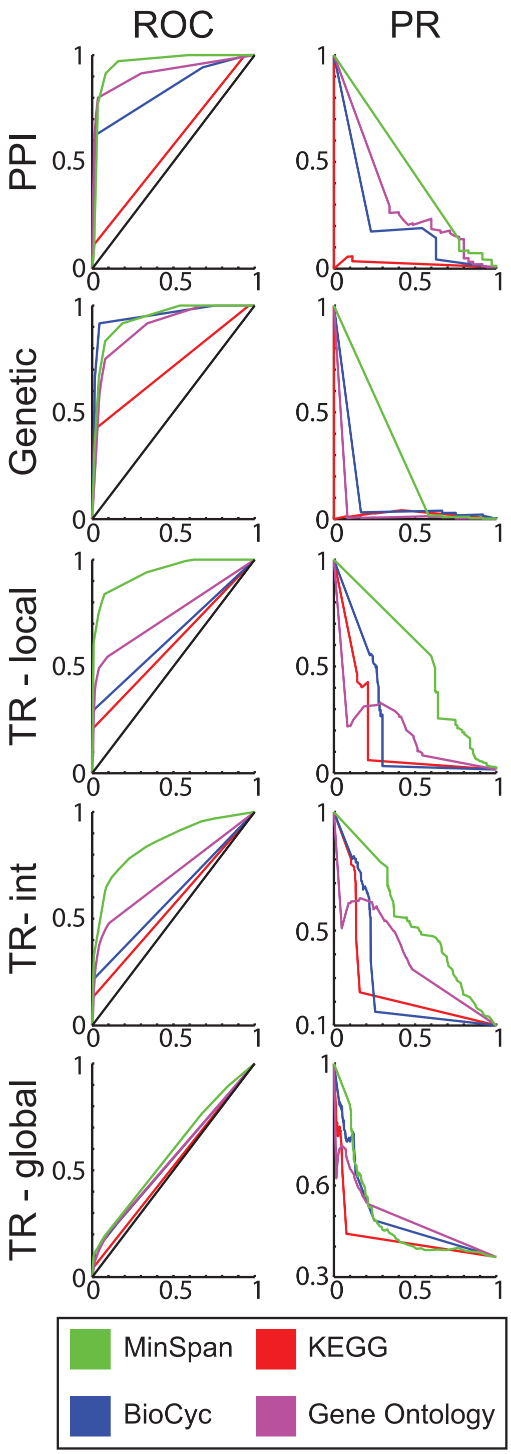

Figure S8: Receiver operating characteristic (ROC) and precision-recall (PR) curves for correlation analysis comparing metabolic pathways (MinSpan, BioCyc, and KEGG) and metabolic gene classifications (Gene Ontology) versus the known underlying biomolecular interactions (protein-protein, positive genetic, and transcriptional regulation). These curves are for the subset of interactions covered by MinSpan.

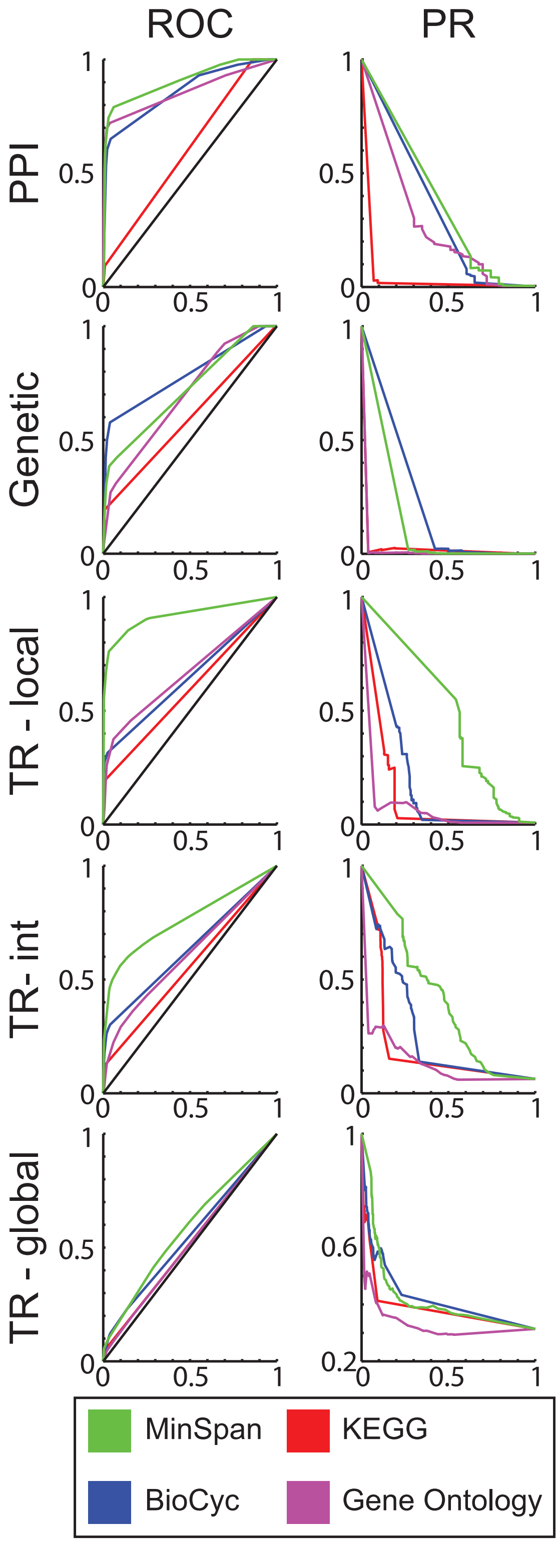

Figure S9: Receiver operating characteristic (ROC) and precision-recall (PR) curves for correlation analysis comparing metabolic pathways (MinSpan, BioCyc, and KEGG) and metabolic gene classifications (Gene Ontology) versus the known underlying biomolecular interactions (protein-protein, positive genetic, and transcriptional regulation). These curves are for the union of all interactions covered by KEGG, BioCyc, Gene Ontology, and MinSpan.

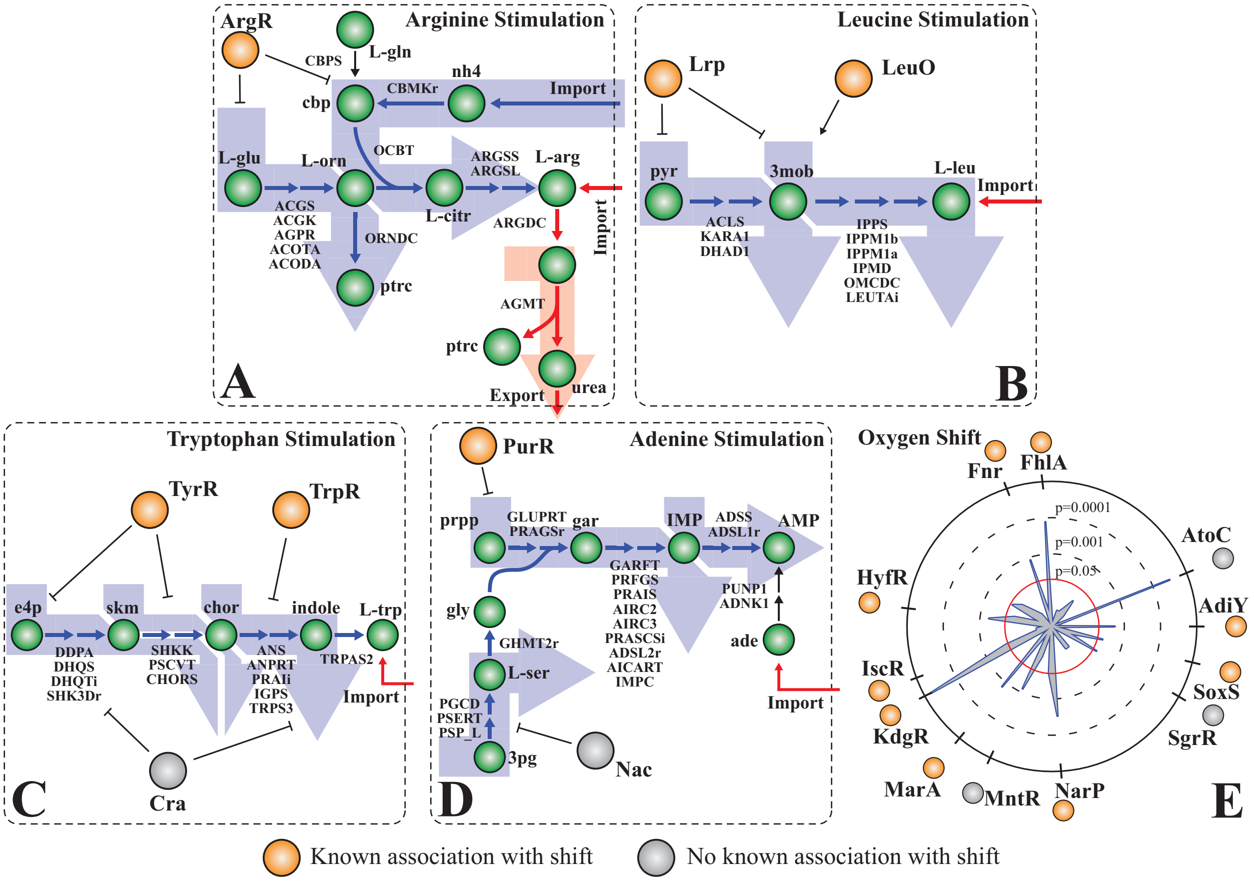

Figure S10: Transcription factor activities can be predicted based on the *E. coli* metabolic network and its MinSpan pathways. **(A)** Projecting sample reaction flux states into MinSpan pathways elucidates specific pathways that are significantly changed and their associated transcription factors. Small arrows represent reactions, while thick arrows represent portions of MinSpan pathways. Activated reactions or pathways during stimulation are indicated in red, repressed in blue. For L-arginine stimulation, 4 pathways and 3 TFs are changed, including ArgR. **(B)** For L-leucine stimulation, 2 pathways and 2 TFs are changed: Lrp and LeuO. **(C)** For L-tryptophan stimulation, 4 pathways and 3 TFs are changed, including TrpR and TyrR. **(D)** For adenine stimulation, 5 pathways and 4 TFs are changed, including PurR. **(E)** For oxygen shift, 70 pathways and 54 TFs are changed. 12 TFs are significantly enriched including Fnr. Oxygen associated TFs are shown in yellow. Reaction and metabolite abbreviations are provided at http://bigg.ucsd.edu.

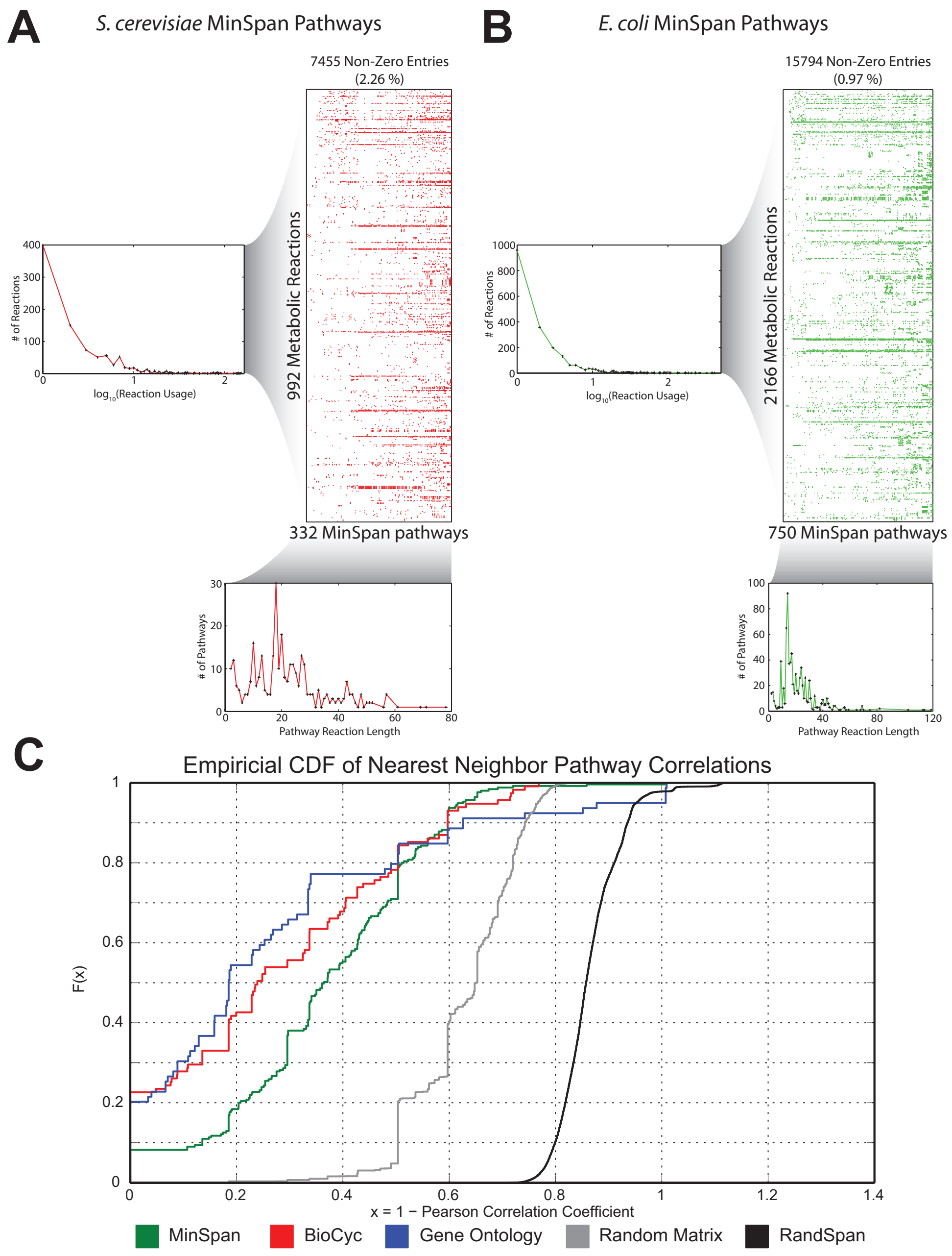

Figure S11: Comparison of conservation of the genome-scale pathways of metabolism in **(A)** *S. cerevisiae* and **(B)** *E. coli*. The MinSpan pathway matrices differ in reaction number (992 vs 2166 metabolic reactions), number of pathways (332 vs 750), and overall sparsity (2.26% and 0.97%). However, the distribution of the number of reactions per pathway and the usage of reactions across the pathways is similar. Most of the pathways contain around 20 reactions. Most reactions are used only once or a few times across all the MinSpan pathways. **(C)** The MinSpan pathways are conserved, based on Pearson correlation, across the two species based on gene products. However, the similarity between pathways is less than human-defined pathways (BioCyc and Gene Ontology).

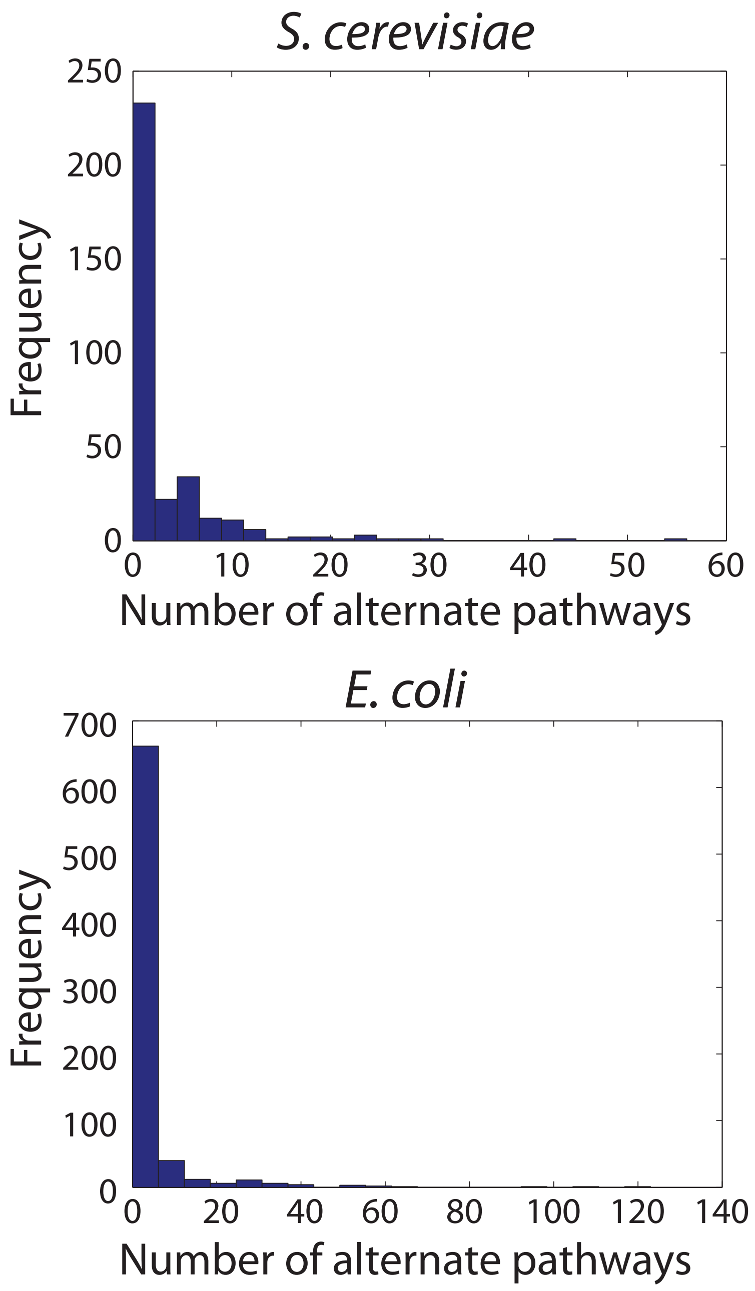

Figure S12: Histogram of the number of alternate pathways for each vector of the MinSpan matrices of *S. cerevisiae* (iMM904) and *E. coli* (iJO1366). The majority of pathways have no alternates or very few (< 5). A few pathways have many alternates.

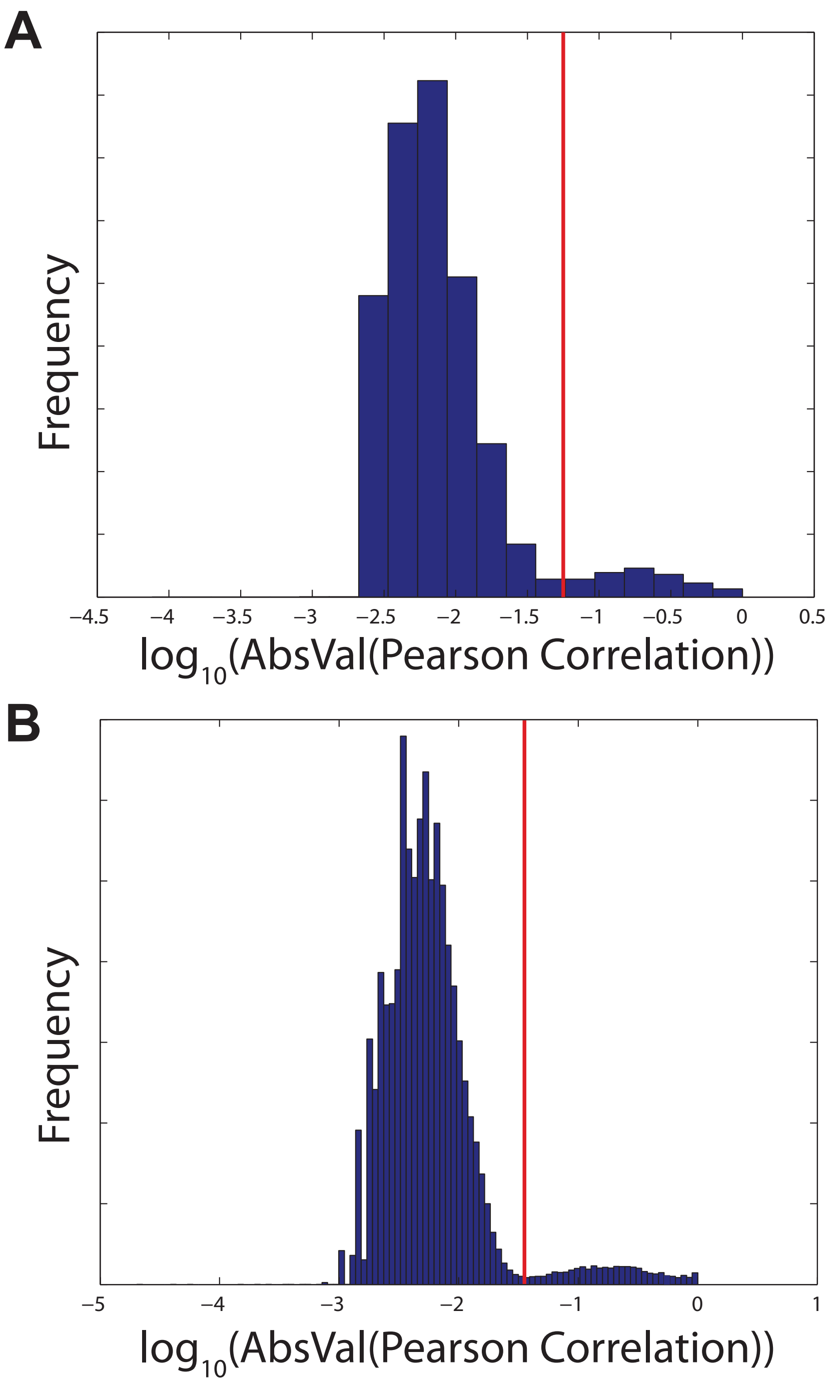

Figure S13: The Connection Specificity Index (CSI) was used to define the correlation and specificity between two pathways to determine their similarity. An empirically derived threshold is needed to make sure that the "noise" from correlation analysis is subtracted. Typically, a threshold of 0.05 is used. For a more robust analysis, we inspected the total distribution of Pearson correlations (log10, absolute value) to determine our thresholds. A threshold (red line) was chosen in order to remove the majority of noise for (A) *S. cerevisiae*, and (B) *E. coli*.

References

Aguilera, L., Campos, E., Gimenez, R., Badia, J., Aguilar, J., and Baldoma, L. (2008). Dual role of LldR in regulation of the lldPRD operon, involved in L-lactate metabolism in Escherichia coli. Journal of bacteriology *190*, 2997-3005.

Amador-Noquez, D., Feng, X.J., Fan, J., Roquet, N., Rabitz, H., and Rabinowitz, J.D. (2010). Systems-level metabolic flux profiling elucidates a complete, bifurcated tricarboxylic acid cycle in *Clostridium acetobutylicum*. J Bacteriol *192*, 4452-4461.

Bodenmiller, D.M., and Spiro, S. (2006). The yjeB (nsrR) gene of Escherichia coli encodes a nitric oxide-sensitive transcriptional regulator. Journal of bacteriology *188*, 874-881.

Cho, B.K., Federowicz, S., Park, Y.S., Zengler, K., and Palsson, B.O. (2012). Deciphering the transcriptional regulatory logic of amino acid metabolism. Nat Chem Biol *8*, 65-71.

Cho, B.K., Federowicz, S.A., Embree, M., Park, Y.S., Kim, D., and Palsson, B.O. (2011). The PurR regulon in Escherichia coli K-12 MG1655. Nucleic acids research *39*, 6456-6464.

Compan, I., and Touati, D. (1994). Anaerobic activation of arcA transcription in Escherichia coli: roles of Fnr and ArcA. Mol Microbiol *11*, 955-964.

D'Autreaux, B., Touati, D., Bersch, B., Latour, J.M., and Michaud-Soret, I. (2002). Direct inhibition by nitric oxide of the transcriptional ferric uptake regulation protein via nitrosylation of the iron. Proceedings of the National Academy of Sciences of the United States of America *99*, 16619-16624.

Demple, B. (1996). Redox signaling and gene control in the Escherichia coli soxRS oxidative stress regulon--a review. Gene *179*, 53-57.

Gunsalus, R.P., Kalman, L.V., and Stewart, R.R. (1989). Nucleotide sequence of the narL gene that is involved in global regulation of nitrate controlled respiratory genes of Escherichia coli. Nucleic acids research *17*, 1965-1975.

Hryniewicz, M.M., and Kredich, N.M. (1994). Stoichiometry of binding of CysB to the cysJIH, cysK, and cysP promoter regions of Salmonella typhimurium. Journal of bacteriology *176*, 3673-3682.

Ikeda, J.S., Janakiraman, A., Kehres, D.G., Maguire, M.E., and Slauch, J.M. (2005). Transcriptional regulation of sitABCD of Salmonella enterica serovar Typhimurium by MntR and Fur. Journal of bacteriology *187*, 912-922.

Izu, H., Adachi, O., and Yamada, M. (1997). Gene organization and transcriptional regulation of the gntRKU operon involved in gluconate uptake and catabolism of Escherichia coli. J Mol Biol *267*, 778-793.

Jardine, O., Gough, J., Chothia, C., and Teichmann, S.A. (2002). Comparison of the small molecule metabolic enzymes of *Escherichia coli* and *Saccharomyces cerevisiae*. Genome research *12*, 916-929.

Kasahara, M., Nakata, A., and Shinagawa, H. (1992). Molecular analysis of the Escherichia coli phoP-phoQ operon. Journal of bacteriology *174*, 492-498.

Keseler, I.M., Collado-Vides, J., Santos-Zavaleta, A., Peralta-Gil, M., Gama-Castro, S., Muniz-Rascado, L., Bonavides-Martinez, C., Paley, S., Krummenacker, M., Altman, T.*, et al.* (2011). EcoCyc: a comprehensive database of Escherichia coli biology. Nucleic acids research *39*, D583-590.

Kurnasov, O.V., Polanuyer, B.M., Ananta, S., Sloutsky, R., Tam, A., Gerdes, S.Y., and Osterman, A.L. (2002). Ribosylnicotinamide kinase domain of NadR protein: identification and implications in NAD biosynthesis. Journal of bacteriology *184*, 6906-6917.

Martin, R.G., Gillette, W.K., Martin, N.I., and Rosner, J.L. (2002). Complex formation between activator and RNA polymerase as the basis for transcriptional activation by MarA and SoxS in Escherichia coli. Mol Microbiol *43*, 355-370.

McNicholas, P.M., and Gunsalus, R.P. (2002). The molybdate-responsive Escherichia coli ModE transcriptional regulator coordinates periplasmic nitrate reductase (napFDAGHBC) operon expression with nitrate and molybdate availability. Journal of bacteriology *184*, 3253-3259.

Mo, M.L., Palsson, B.O., and Herrgard, M.J. (2009). Connecting extracellular metabolomic measurements to intracellular flux states in yeast. BMC systems biology *3*, 37.

Murray, E.L., and Conway, T. (2005). Multiple regulators control expression of the Entner-Doudoroff aldolase (Eda) of Escherichia coli. Journal of bacteriology *187*, 991-1000.

Orth, J.D., Conrad, T.M., Na, J., Lerman, J.A., Nam, H., Feist, A.M., and Palsson, B.O. (2011). A comprehensive genome-scale reconstruction of Escherichia coli metabolism--2011. Molecular systems biology *7*, 535.

Orth, J.D., Fleming, R.M., and Palsson, B.O. (2009). Reconstruction and Use of Microbial Metabolic Networks: the Core Escherichia coli Metabolic Model as an Educational Guide. In EcoSal - Escherichia coli and Salmonella Cellular and Molecular Biology, P.D. Karp, ed. (Washington, DC, ASM Press).

Palsson, B.O. (2006). Systems biology: properties of reconstructed networks (New York, Cambridge University Press).

Quail, M.A., and Guest, J.R. (1995). Purification, characterization and mode of action of PdhR, the transcriptional repressor of the pdhR-aceEF-lpd operon of Escherichia coli. Mol Microbiol *15*, 519-529.

Ramseier, T.M., Bledig, S., Michotey, V., Feghali, R., and Saier, M.H., Jr. (1995). The global regulatory protein FruR modulates the direction of carbon flow in Escherichia coli. Mol Microbiol *16*, 1157-1169.

Salmon, K., Hung, S.P., Mekjian, K., Baldi, P., Hatfield, G.W., and Gunsalus, R.P. (2003). Global gene expression profiling in Escherichia coli K12. The effects of oxygen availability and FNR. The Journal of biological chemistry *278*, 29837-29855.

Sasse-Dwight, S., and Gralla, J.D. (1988). Probing the Escherichia coli glnALG upstream activation mechanism in vivo. Proceedings of the National Academy of Sciences of the United States of America *85*, 8934-8938.

Sauter, M., Bohm, R., and Bock, A. (1992). Mutational analysis of the operon (hyc) determining hydrogenase 3 formation in Escherichia coli. Mol Microbiol *6*, 1523-1532.

Schlensog, V., and Bock, A. (1990). Identification and sequence analysis of the gene encoding the transcriptional activator of the formate hydrogenlyase system of Escherichia coli. Mol Microbiol *4*, 1319-1327.

Schwartz, C.J., Giel, J.L., Patschkowski, T., Luther, C., Ruzicka, F.J., Beinert, H., and Kiley, P.J. (2001). IscR, an Fe-S cluster-containing transcription factor, represses expression of Escherichia coli genes encoding Fe-S cluster assembly proteins. Proceedings of the National Academy of Sciences of the United States of America *98*, 14895-14900.

Simon, G., Mejean, V., Jourlin, C., Chippaux, M., and Pascal, M.C. (1994). The torR gene of Escherichia coli encodes a response regulator protein involved in the expression of the trimethylamine N-oxide reductase genes. Journal of bacteriology *176*, 5601-5606.

Unden, G., and Dunnwald, P. (2008). Chapter 3.2.2 The Aerobic and Anaerobic Respiratory Chain of Escherichia coli and Salmonella enterica: Enzymes and Energetics. In EcoSal-Escherichia coli and Salmonella: Cellular and Molecular Biology, A. Bock, R. Curtiss III, J.B. Kaper, P.D. Karp, F.C. Neidhardt, T. Nystrom, J.M. Slauch, C.L. Squires, and U. D., eds. (Washington, DC, ASM Press).

Unden, G., and Trageser, M. (1991). Oxygen regulated gene expression in Escherichia coli: control of anaerobic respiration by the FNR protein. Antonie Van Leeuwenhoek *59*, 65-76.

Yang, B., Gerhardt, S.G., and Larson, T.J. (1997). Action at a distance for glp repressor control of glpTQ transcription in Escherichia coli K-12. Mol Microbiol *24*, 511-521.

Yeung, M., Thiele, I., Palsson, B. Ø. (2007). Estimation of the Number of Extreme Pathways for Metabolic Networks. BMC bioinformatics *8*.

Zheng, M., Aslund, F., and Storz, G. (1998). Activation of the OxyR transcription factor by reversible disulfide bond formation. Science (New York, NY *279*, 1718-1721.
